# ER Stress-Mediated Apoptosis in a New Mouse Model of Osteogenesis imperfecta

**DOI:** 10.1371/journal.pgen.0040007

**Published:** 2008-02-01

**Authors:** Thomas S Lisse, Frank Thiele, Helmut Fuchs, Wolfgang Hans, Gerhard K. H Przemeck, Koichiro Abe, Birgit Rathkolb, Leticia Quintanilla-Martinez, Gabriele Hoelzlwimmer, Miep Helfrich, Eckhard Wolf, Stuart H Ralston, Martin Hrabé de Angelis

**Affiliations:** 1 Institute of Experimental Genetics, Helmholtz Zentrum München, German Research Center for Environmental Health, Neuherberg, Germany; 2 Institute of Molecular Animal Breeding and Biotechnology, Ludwig-Maximilians University, Munich, Germany; 3 Institute of Pathology, Helmholtz Zentrum München, German Research Center for Environmental Health, Neuherberg, Germany; 4 Department of Medicine and Therapeutics, University of Aberdeen, Institute of Medical Sciences, Foresterhill, Aberdeen, United Kingdom; 5 Molecular Medicine Centre, University of Edinburgh, Western General Hospital, Edinburgh, United Kingdom; Stanford University School of Medicine, United States of America

## Abstract

Osteogenesis imperfecta is an inherited disorder characterized by increased bone fragility, fractures, and osteoporosis, and most cases are caused by mutations affecting the type I collagen genes. Here, we describe a new mouse model for Osteogenesis imperfecta termed *Aga2* (abnormal gait 2) that was isolated from the Munich N-ethyl-N-nitrosourea mutagenesis program and exhibited phenotypic variability, including reduced bone mass, multiple fractures, and early lethality. The causal gene was mapped to Chromosome 11 by linkage analysis, and a C-terminal frameshift mutation was identified in the *Col1a1* (procollagen type I, alpha 1) gene as the cause of the disorder. *Aga2* heterozygous animals had markedly increased bone turnover and a disrupted native collagen network. Further studies showed that abnormal proα1(I) chains accumulated intracellularly in *Aga2*/+ dermal fibroblasts and were poorly secreted extracellularly. This was associated with the induction of an endoplasmic reticulum stress-specific unfolded protein response involving upregulation of *BiP*, *Hsp47*, and *Gadd153* with caspases-12 and −3 activation and apoptosis of osteoblasts both in vitro and in vivo. These studies resulted in the identification of a new model for *Osteogenesis imperfecta,* and identified a role for intracellular modulation of the endoplasmic reticulum stress-associated unfolded protein response machinery toward osteoblast apoptosis during the pathogenesis of disease.

## Author Summary

Osteogenesis imperfecta (OI) is a heterogeneous collection of connective tissue disorders typically caused by mutations in the *COL1A1/2* genes that encode the chains of type I collagen, the principle structural protein of bone. Phenotypic expression in OI depends on the nature of the mutation, causing a clinical heterogeneity ranging from a mild risk of fractures to perinatal lethality. Here, we describe a new OI mouse model with a dominant mutation in the terminal C-propeptide domain of Col1a1 generated using the N-ethyl-N-nitrosourea (ENU) mutagenesis strategy. Heterozygous animals developed severe-to-lethal phenotypes that were associated with endoplasmic reticulum stress, and caspases-12 and −3 activation within calvarial osteoblasts. We provide evidence for endoplasmic reticulum stress–associated apoptosis as a key component in the pathogenesis of disease.

## Introduction

Mutations in type I collagen genes (*COL1A1/2*) typically lead to Osteogenesis imperfecta (OI), the most common heritable cause of skeletal fractures and bone deformation in humans [1]. OI is classified into eight human subtypes, and to date greater than 500 human *COL1A1* mutations have been reported representing a clinical heterogeneity dictated by the complex array of mutations. Recently, novel molecules and loci apart from classic type I collagens have been implicated in both murine [2] and human [3–5] alternative recessive forms of OI, thus expanding the genetic heterogeneity.

Type I collagen is the most common ubiquitously expressed fibrous protein in the extracellular matrix (ECM) of connective tissues with both biomechanical and physiological functions [6]. Type I collagen initially exists as a procollagen precursor with NH_2_- and COOH-terminal propeptide domains with distinct roles. Type I procollagen molecules consist of three polypeptide coiled subunit chains (two proα1(I) and one proα2(I) chain) that self-associate in the endoplasmic reticulum (ER), and require a highly coordinated post-translational regulation. The helical procollagens are deposited into the extracellular space, proteolytically cleaved, and then organized into highly ordered collagen fibrils covalently cross-linked to increase tensile strength and rigidity. Apart from its biomechanical properties, type I collagen stores key factors for remodeling maintenance, and acts as an adhesive substrate with cellular receptors and other matricellular components along its major ligand binding regions [7]. These properties regulate complex intracellular signal transduction pathways for tissue remodeling and repair, immune response, polarization, migration, proliferation, differentiation, and cell survival within various cellular contexts [8].

Based on detailed radiographic, molecular genetic and morphological analyses, structural collagen mutations are likely associated with lethal (type II) and moderate (types III and IV) forms of OI [1]. Type II OI represents the most severe form of the disease accounting for ∼20 % of cases, and the heterogeneous clinical and biochemical aspects have been described [9]. Most OI-II probands acquire *new* dominant mutations in *COL1A1/2*, and a low frequency recurrence risk can be the result of gonadal mosaicism in one of the parents [10]. OI-II-related mutations behave in a dominant negative manner as the mutant proα(I) proteins affect wild-type chain registration and formation due to the polymeric nature of assembly, impairing overall ECM stoichiometry and integrity. Typically in OI-II cells, abnormal trimers are poorly passaged through the secretory pathway, and are more vulnerable to degradation and overmodification, thus the cytoprotective unfolded protein response (UPR) is concordantly instigated [11–13].

Despite the observation of machinery involving BiP chaperones that might target most defective procollagens for subsequent proteasomal degradation in OI-II cells [13], the direct activation of apoptosis has not been shown to date. Here, we provide the first report on the isolation, cloning and characterization of the skeletal phenotype in a novel ENU-induced mouse line for human OI affecting the terminal C-propeptide region of Col1a1. Furthermore, we provide evidence of caspases-12 and −3-mediated apoptosis in *Aga2* osteoblasts as a critical step toward increased cell death, thus broadening our molecular insight into OI.

## Results

### Identification and Lethality of *Aga2*/+

The original *Aga2*/+ mouse was identified in the Munich ENU dominant mutagenesis screen [14], and displayed an abnormal gait due to deformity of the hind limbs at five weeks of age among the F_1_ mutagenized progeny. *Aga2*/+ males have general reproductive success and the mutation segregated in a dominant manner with complete penetrance (Table 1). *Aga2*/+ females produced smaller litter sizes due to a reduction in body size. A large subset of *Aga2*/+ animals succumbed to postnatal lethality (Table 1), and featured severe bone deformities and fractures. *Aga2*/+ *inter se* mating yielded non-Mendelian ratios for dominant inheritance suggesting embryonic lethality. Various embryonic stages were investigated to determine gestational arrest in homozygotes, which was estimated to occur around embryonic day 9.5 post coitum (data not shown).

**Table 1:**
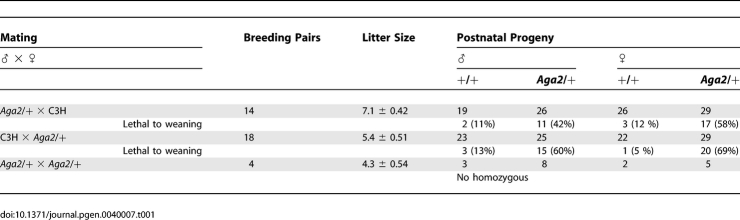
Mode of Inheritance and Postnatal Lethality

### Positional Cloning and Characterization of the *Aga2* Mutation

**Figure 1:**
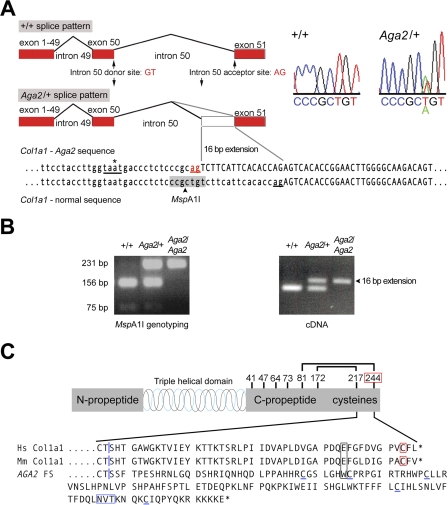
The C-Propeptide *Col1a1* ^Aga2^ Mutation (A) Mutation scheme. In *Aga2*, splicing occurs at the new splice acceptor site (ag, highlighted red). The taat domain (underlined) harbors a putative spliceosome branch site (*). (B) Genotyping and presence of the extended transcript. (C) Alignment of human and murine Col1a1 terminal C-propeptides and prediction of the *Aga2* frameshifted sequence. The start of mouse exon 51 (52 in human) is marked by a blue line. The frameshifted cysteine (C244) is boxed in red, newly introduced cysteines are underlined in blue; the potential *N*-linked oligosaccharide (*CHO*) attachment site is boxed in blue. Start of the human frameshift mutation described by Willing et al. [30] is boxed in black.

Genetic mapping of the *Aga2* locus was performed following the standard outcross-backcross breeding strategy. The mutation was mapped to a 700 kb domain on Chromosome 11 (Figure S1). Within the *Aga2* candidate region, two skeletal genes were identified, *Col1a1* (procollagen type I, alpha1) and *Chad* (chondroadherin). Both candidate genes were sequenced and a novel T to A transversion mutation within intron 50 of *Col1a1* was identified (Figure 1A). The *Aga2* substitution generated a novel 3′ splice acceptor site and predicted a terminal frameshift beyond the endogenous stop (Figure 1C). On the transcript level, cDNA from homozygous embryos revealed a 16 bp expanded transcript, whereas in heterozygous embryos equal levels of two transcripts are present (Figure 1B). We further confirmed the mutation at the genomic level. The T to A exchange disrupted the endogenous MspA1I restriction site, generating a cleavage-resistant allele in *Aga2/+* samples (Figure 1B).

The C-terminal portion of human, and murine as well as *Aga2* mutant Col1a1 was aligned highlighting specific residues of importance (Figure 1C). Of interest, the most terminal conserved cysteine C244 (aa 1451) was ablated, and 48 endogenous amino acids including the stop were frameshifted, concomitantly predicting 90 new amino acids beyond the termination position. The *Aga2* frameshift begins 56 amino acids away from the chain selectivity sequence [15]. Additionally, five new cysteines and a potential *N*-linked oligosaccharide (*CHO*) attachment site were introduced. The secondary structure of the *Aga2* product was predicted to remove the hydrophobicity of the short loop formed by the last intra-chain disulphide bond (data not shown).

### *Aga2* Skeletal and Growth Phenotypes

**Figure 2:**
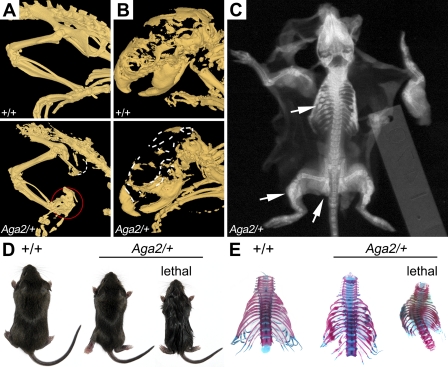
*Aga2*/+ Skeletal and Growth Abnormalities (A) Lateral μCT views of 12-wk-old male skeletons highlight the axial vertebrae, pelvis (white trace), and hind limb (encircled) defects in *Aga2*/+ mice. (B) Cranial features of a 16-d-old lethal *Aga2*/+ mouse. (C) Skeletal radiograph of a 16-d-old lethal *Aga2*/+ animal. Provisional-bony rib and long bone calluses and deteriorated pelvis depicted with arrows. (D) Severe growth defects in *Aga2*/+ animals. (E) Skeletal preparations.

Among all *Aga2*/+ animals, the gross skeletal phenotype was discernable starting between days 6 - 11 after birth. Heterozygous animals that survived to adulthood were classified as moderately-to-severely affected and displayed the hallmark dystrophic limb(s), long bone and pelvis fractures, reduced body size, and generalized decreases in DXA-based bone parameters (Figure 2 and Table S1; data were collected from the German Mouse Clinic dysmorphology primary screen [16]). Lethal animals developed thin calvaria, hemorrhaging at joint cavities and intracranial sites, scoliosis, provisional rib and long bone calluses and deformities, body size deficit, *pectus excavatum*, gasping, cyanosis, platyspondyly, edema of the eyes, greasy skin, and eczema (Figure 2 and data not shown). Comminuted fractures incurred signifying the severe brittleness of *Aga2*/+ bones accompanied by many repair blastemas characteristic of a type II OI phenotype.

The effect of the mutation on volumetric bone mineral density (vBMD) and content (vBMC) was evaluated in adult mice using in vivo peripheral quantitative computed tomography (pQCT; Table S2). In *Aga2*/+ distal femora, trabecular and cortical vBMD as well as cortical vBMC were substantially decreased compared to controls. In contrast, the trabecular vBMC was unchanged due to enlarged medullar areas (suggesting elevated resorption in *Aga2*/+). Collectively, these results suggest potential defects in mineralization and/or bone formation in *Aga2*/+ mice.

### Clinical Chemical and Hormone Analysis

Several serum biochemical and hormonal markers were evaluated to detect possible bone metabolic disturbances (Table S3). Total alkaline phosphatase (ALP) and osteocalcin levels were significantly increased in both sexes in *Aga2*/+ compared to littermate controls. In addition, circulating TRACP 5b (an osteoclast marker) was significantly elevated in *Aga2*/+. No changes in inorganic phosphate levels were observed (data not shown). *Aga2*/+ animals depicted a significant increase in PTH levels. Furthermore, *Aga2*/+ mice yielded a distinct increase in calcitonin, which was more pronounced in females. Taken together, the significantly increased bone formation and resorption markers indicate that in *Aga2*/+ mice metabolic bone turnover is elevated. This was further supported by histomorphometric analysis of bone formation and resorption showing increased numbers of endogenous osteoblasts and osteoclasts, an increased bone formation rate (BFR) and a reduced mineral apposition rate (MAR) in *Aga2/+* bone samples (Figures S2, S3 and Table S4).

### Altered Cellular and Collagen Structures in *Aga2*/+

**Figure 3:**
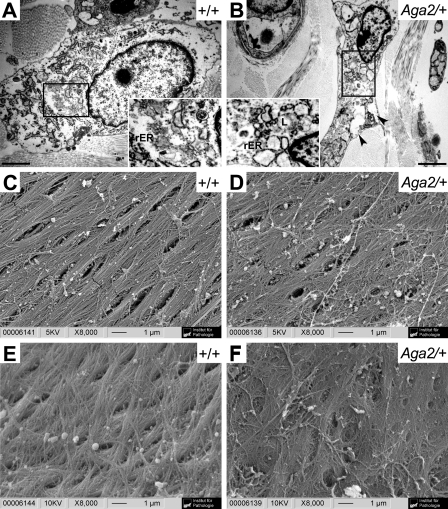
Electron Microscopical Findings in Dermal Fibroblasts and Cortical Bone (A,B) TEM micrographs of wild-type and *Aga2*/+ fibroblasts. The rough endoplasmatic reticulum (rER) is engorged in *Aga2*/+ cells (bars, 2 μm for main, 1 μm for inset). Increased intracellular empty vacuoles (arrow heads) and lysomes (L) were present in *Aga2*/+. (C–F) SEMs of femoral endosteal surfaces reveal differences in organization of the collagen matrix (C,D) Outer cortical surface. (E,F) Inner cortical surface.

Fibroblast and type I collagen features in *Aga2*/+ connective tissues were evaluated by transmission and scanning electron microscopy (TEM and SEM). *Aga2*/+ dermis contained more heterogeneous populations of fibroblasts with smaller nuclei, aberrant dilated electron-dense ERs, lysosomes and empty autophagic-like vacuoles interspersed throughout the cells (Figure 3A and 3B). *Aga2*/+ ERs appeared contiguous with secretory vesicles signifying the formation of ER associated compartments. SEM studies on cortical nanostructure of *Aga2*/+ samples depicted a less-parallel, less-densely packed network of collagen bundles when compared to controls (Figure 3C–3F).

**Figure 4:**
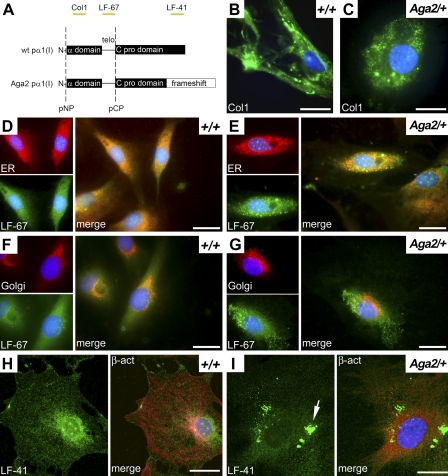
Immunofluorescence Microscopy of Type I and proα1(I) Collagen in *Aga2/+* Primary Dermal Fibroblasts (A) Scheme depicting probe-specificity toward various isoforms (pN/CP, procollagen N/C-proteinase; telo, telopeptide domain). (B,C) Mature type I collagen. (D-G) Proα1(I) double immunofluorescence using antiserum LF-67, protein disulphide isomerase (PDI), and golgi phosphoprotein 4 (GOLPH4) for detection of the ER and *cis*-Golgi apparatus, respectively. The combined panels (E) and (G) show proα1(I) chain retention within the ER, not in the *cis*-Golgi respectively, when compared to controls (D,F). It is speculated that aberrant procollagen molecules aggregate into vesicular structures (E) (merged green dots), which may be part of the proteasome. (H,I) Proα1(I) double immunofluorescence using antiserum LF-41 and β-actin antibody for the cytoskeleton (arrow, aberrant procollagen). All nuclei were counterstained with DAPI. Bars, 25 μm.

Procollagen trafficking was assessed in immunofluorescence studies using dermal fibroblasts (Figure 4). To uncover mature type I collagen integrity we used an antibody that was directed against the triple helical domain and was also capable of detecting procollagens (Figure 4A). Wild-type cells depicted both intracellular and intact extracellular surface staining (Figure 4B). In *Aga2*/+ cells, the intact type I collagen surface stain was largely reduced while intracellularly retaining procollagen molecules were stained (Figure 4C). Double immunofluorescence staining of proα1(I) chains was performed using antiserum LF-67 (Figure 4A), protein disulphide isomerase (PDI), and Golgi phosphoprotein 4 (GOLPH4) for detection of the ER and *cis*-Golgi apparatus, respectively. Merged stainings showed proα1(I) chain retention within the ER (Figure 4E) but not in the *cis*-Golgi (Figure 4G), when compared to controls (Figure 4D and 4F). It is speculated that aberrant procollagen molecules aggregate into vesicular structures (Figure 4E, merged green dots), which may be part of the proteasome. To further clarify the intracellular abnormalities double immunofluorescence staining of proα1(I) chains was performed using antiserum LF-41 and β-actin antibody for the cytoskeleton. The LF-41 epitope resides within the wild-type terminal C-propeptide overlapping segment of the *Aga2* frameshifted region and captures only molecules that contain chains with the normal C-propeptide (Figure 4A). Using antiserum LF-41, control fibroblasts depict dynamic intracellular procollagen and isoform trafficking to the infoldings of the plasma membrane (Figure 4H). Contrarily, *Aga2*/+ fibroblasts retained intracellular (wild-type) procollagen molecules at near peri-nuclear regions (Figure 4I, arrow). Surface staining in both wild type and *Aga2*/+ cells was limited given that the LF-41 antiserum was unable to discriminate mature, proteolysed type I collagen in the ECM. Based on our results, the mutation affected proα1(I) chain processing by blocking its ER-to-Golgi anterograde transport, thus inhibiting vesicular exocytosis to the matrix.

### Altered Ex Vivo Osteogenic Activity in *Aga2*/+ Calvarial Culture System

**Figure 5:**
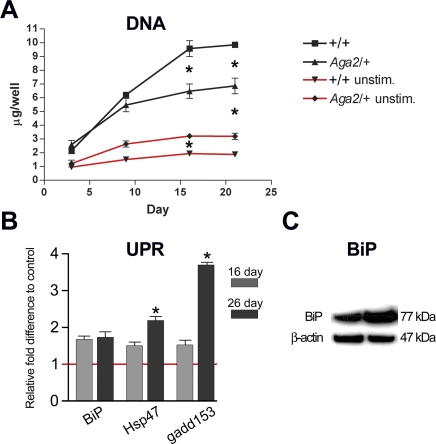
Anomalies in *Aga2/+* Osteoblast Growth and Induction of Cytoprotective Unfolded Protein Response (A) Growth curve analysis of +/+ and *Aga2*/+ primary calvarial culture system. (B) UPR, qPCR analysis of stimulated calvarial OBs. Values represent means ± SEM, where *n* = 4–5 experiments (*p < 0.01 Student *t*-test). (C) Western analysis of BiP using primary OBs.

OB metabolism and function were characterized utilizing the primary calvarial culture system. We determined the growth curve of *Aga2*/+ primary OBs and observed a limited saturation density within the stimulated samples (Figure 5A) that was accompanied by a relative increase in ALP levels until day 26 (Figure S4A). Also, *Aga2*/+ total protein and mitochondrial reductase activity levels were significantly increased (Figure S4B). Consistent with our immunofluorescence studies there was a clear reduction in the total amount of secreted acid-soluble collagens tested in *Aga2*/+ media (Figure S4B). Of note, unstimulated *Aga2*/+ OBs depicted no significant change in cellular protein and metabolic activity levels (data not shown).

To address the functional defects in *Aga2*/+ OBs, nodular formation, growth and binding capacities were studied over time (Figure S4C). The formation of bone-like nodules was persistently reduced between days 9 and 16 in *Aga2*/+. In addition, the average area of individual *Aga2*/+ nodules was reduced compared to controls. Lastly, a 3.2 fold comparative increase in nodular dye-binding capacity was observed in *Aga2*/+ (Figure S4C). Taken together, these results demonstrate clear functional and metabolic disturbances in *Aga2*/+ OBs.

### Initiation of ER Stress-Induced Apoptosis in *Aga2*/+ OBs

To show involvement of key regulators of ER stress response pathways, we performed qPCR studies using primary calvarial OBs (Figure 5B). At days 16 and 26 in culture (11 and 21 days after induction, respectively), expression of the molecular chaperones genes *Hspa5* (also known as *BiP/GRP78*) and *Serpinh1* (also known as *Hsp47*) was upregulated ∼1.5 – 2.2 folds above control. The expression of *Ddit3* (also known as *Gadd153/chop*), a transcription factor involved in the induction of apoptosis, was ∼3.7 folds higher in *Aga2*/+ cells at day 26 compared to control levels. To confirm the induction of cytoprotective unfolded protein response (UPR) in *Aga2*/+ cells, Western analysis of BiP protein expression was performed using primary calvarial OBs. As shown in Figure 5C BiP protein levels were increased compared to controls.

**Figure 6:**
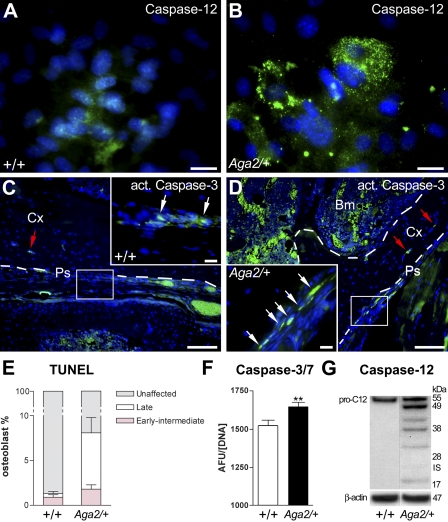
Initiation of Apoptosis in *Aga2*/+ (A,B) Caspase-12 immunofluorescence of 16-d-old stimulated OBs. (C,D) Activated caspase-3 labeling of 12-wk-old distal femur depicting dying cells within the periosteum (white arrows) and cortical bone (red arrows). Nuclei were stained with DAPI. (E) Quantification of TUNEL stained 16-d-old stimulated calvarial OBs. (F) Caspase-3/7 OB activity was measured via a fluorometric substrate assay. (G) Western analysis of procaspase-12 using primary OBs. Results are presented as mean ± SEM (*p = 0.0002 ANOVA, and **p = 0.04 Student *t*-test), where *n* = 4 independent experiments. Bars, 50 μm in (A) and (B), 100 μm in (C) and (D) main panels, 20 μm in (C) and (D) insets. Arbitrary fluorescent unit (AFU), bone marrow (Bm), cortical bone (Cx), low-present isoform (IS), periosteum (Ps).

Within the context of ER-induced stress we investigated cell apoptosis. The *Aga2* mutation led to a combined relative increase in the number of early-late stage TUNEL-positive picnotic OBs compared to control (Figure 6E). At 16 days in culture, *Aga2*/+ preparations depicted a 10 % relative increase in the number of OBs, which contained aberrant caspase-12-immunoreactivity (Figure 6A and 6B; 3.3 ± 0.9 % +/+ and 12.9 ± 1.9 % *Aga2*/+; *p* = 0.0007 Student *t*-test). For specificity, proteolytic processing of procaspase-12 was evident only in *Aga2*/+ samples via Western analysis (Figure 6G). Caspase-3/7 activation was independently confirmed depicting a significant 9 % comparative increase in activity in *Aga2*/+ OBs (Figure 6F). Lastly, activated caspase-3 immunoreactivity was observed within femoral periosteum (Figure 6C and 6D). *Aga2*/+ tissue contained elevated relative numbers of activated caspase-3-positive OBs (28.9 ± 2.7 % +/+ and 42.6 ± 4.1 % *Aga2*/+; *p* = 0.02 Student *t*-test), suggesting a 14 % comparative basal increase in apoptosis. Also, TUNEL experiments revealed increased numbers of DNA-fragmented OBs within *Aga2*/+ periosteum (data not shown).

## Discussion

ENU mutagenesis is a powerful approach to identify mouse models for human skeletal disorders by generating novel phenotypes. Human mutations affecting the α1/2(I) C-propeptide coding and non-coding regions have been identified less often than other types of mutations and reflect strong variability in clinical outcomes. Here, we provide the first report on the cloning and phenotypic description of the novel mouse line *Aga2*, which bear a chemically induced genetically stable mutation affecting the terminal C-propeptide domain of Col1a1. We provide evidence that abnormal proα1(I) chains accumulated intracellularly and were poorly secreted extracellularly. Furthermore, we show for the first time that this was associated with the induction of an ER stress-specific unfolded protein response (UPR) with caspases 12 and 3 activation and apoptosis of osteoblasts both in vitro and in vivo. The *Aga2* line provides a unique tool for understanding the underlying mechanisms of the incurable debilitating human disease Osteogenesis imperfecta (OI) and the efficacy of novel therapeutic strategies.

### *Aga2*/+ Skeletal Phenotypes

*Aga2*/+ mice feature phenotypic variability ranging from postnatal lethality to moderate-to-severe changes including increased bone fractures, fragility, deformity, osteoporosis, and disorganized trabecular and collagen structures. Compared to the lethal transgenic lines [17,18], the *Aga2*/+ phenotype is stronger and associated with increased lethality, but comparable to the *BrtlIV* knock-in (*Col1a1* ^G349C^) line [19]. The biochemical serum and histomorphometric results of *Aga2*/+ mice indicated an elevated bone turnover that is described similarly for OI patients [20–23]. Our ex vivo studies showed that *Aga2*/+ OBs deposited less collagen matrix, and were overly active, affecting nodule growth and function.

### *Aga2*/+ proα1(I) Chain Defects and Human Case Correlations

The procollagen triple helix proceeds from the carboxyl to amino end, and this association is modulated by correct folding via intra-chain disulphide bonds in the C-propeptide region before chain association and inter-chain disulphide linkage [24,25]. In certain C-propeptide OI-II lethal conditions, the mutated proα1/2(I) chains can associate with normal chains to form secreted triple helical procollagen molecules despite alterations in endogenous disulfide bonds [12,26], while in other lethal cases chain formation with the mutated propeptide is entirely or largely precluded [27,28]. Based on these results, the lethal OI phenotypes are heterogeneously derived from structural and/or cellular metabolic defects.

At the biochemical level, we report significant increased retention of aberrant procollagen molecules within *Aga2*/+ cells, compromising cellular metabolism and the ECM. Moreover, we show that the majority of accumulated chains were wild type in nature. Gel analysis of cDNA showed significant splicing of the new *Aga2* splice site. Although cryptic mRNA transcripts were readily available for translation, unstable mRNA decay and/or anomalies in transport from the nucleus to the cytoplasm due to secondary structure may have mediated low-level translation of mutant chains. The presumptive elongated chain with newly introduced cysteins and disruption of a crucial beta sheet due to the *Aga2* mutation likely destabilized protein conformation, thus affecting preferential chain assembly and proteolysis. Also, a *N*-linked oligosaccharide unit was introduced capable of altering intracellular processing and secretion of procollagens as well [29].

Our findings were consistent with observations made by Willing et al. [30] who investigated a human OI family that harbored a mutation in the 3' end of *COL1A1*, which ablated the intra-chain disulfide cysteine as in the *Aga2* condition. Fitzgerald et al. [13] reported that the fate of mutated unassembled C-propeptide chains from the proband described by Willing et al. were intracellularly degraded in the proteasome. Similar to *Aga2*, it was unclear if the mutated chains ever incorporated into procollagen molecules causing protein suicide and the expected downstream effects [31], or involved an alternative mechanism to influence the clinical outcome.

### ER Stress and Degradation within *Aga2*/+ Cells

Many disorders result from the cell's inability to export mutated proteins and enzymes from the ER, including OI [32,33]. Depending on the nature of the mutation, incorrectly folded proα1/2(I) molecules are managed via multipartite highly regulated sorting pathways in order to retain ER homeostasis and to prevent the secretion of abnormal proteins. The cytoprotective unfolded protein response (UPR) involves the activation of complex regulatory ER stress signaling mechanisms that can either repress protein synthesis or upregulate ER-resident chaperons and other translation regulators [34]. BiP/Grp78, a central regulator of ER function, was shown to bind to proα (I) chains from cell strains, which harbored unique C-propeptide mutations that inhibited chain association [11] and mediated intracellular ER-degradation [12]. Contrarily, mutations that inhibit folding in the triple helical domain of type I procollagen chains do not result in abnormal BiP binding or induction even though the aberrant molecules are retained within the ER [35].

In *Aga2*/+, the presence of dilated ERs and accumulation of aberrant procollagens in the ER strongly support intracellular trafficking defects and degradation within the proximal region of the secretory pathway. We speculate that aberrant procollagen molecules aggregated into vesicular structures (Figure 4E), which are part of the proteasome. Degradation did occur within more distal secretory compartments by the presence of lysosome-like structures [36]. In *Aga2*/+ OB cultures treated with ascorbate, BiP levels were slightly upregulated implicating its constitutive involvement during ER stress. Previous studies from Chessler et al. suggested that in skin fibroblasts from OI patients with mutations in the C-terminal propeptide BiP peaked about 48 hours after exposure to ascorbate and that longer exposure resulted in decreasing levels [11]. As cells studied here were exposed to ascorbate continuously during growth as part of their medium enrichment, we performed Western analysis and confirmed increased BiP protein levels. Hsp47, another molecular chaperone, which is part of the ‘quality control' system for procollagens, is enhanced in certain OI cases [37]. In our studies, the degree of *Hsp47* induction was also elevated. Thus, in *Aga2*/+ both BiP and Hsp47 seemed to choreograph the intracellular regulation of mutant type I procollagens with concomitant induction of apoptosis.

### Ex Vivo and In Vivo Evidence for Increased ER Stress-Induced Apoptosis

Many diseases are associated with either inhibition or increase of apoptosis [34,38], but to date the active involvement of apoptosis during OI pathogenesis is inconclusive. Caspase-12 is a specific mediator of the ER stress-induced UPR within skeletal tissue [39]. In primary *Aga2*/+ OBs, removal of the procaspase-12 adaptor protein-binding domain was evident, and the processing of procaspase-12 demonstrated the ER stress origin of mutated proα1(I) initiator signals. In addition, the presence of increased activated caspase-3 and TUNEL-positive OBs in *Aga2*/+ primary culture and periosteum also implicated a tendency toward apoptosis commitment and cell death, respectively.

Although several models have been proposed for the direct activation of caspase-12 the entire process has yet to be described. The Bcl2 family-proteins are well-established components of the apoptotic machinery, some of which are associated with ER stress pathways. For example, Bim (Bcl2-interacting mediator of cell death) activates caspase-12 via translocating from the dynein-rich ER compartment upon stress [40]. BiP is known to inhibit caspase-mediated cell death by forming complexes with procaspases 7 and 12, and BiP disassociation facilitates procaspase activation [41]. Gadd153/CHOP is a key transcription factor in the regulation of cell growth, differentiation and ER stress-induced apoptosis. Gadd153 induces death by promoting ER client protein load and oxidation via transcription of target genes, which mediate apoptosis, presumably leading to caspase cascades [34]. In our studies, *Gadd153* was highly induced over time, and was presumably triggered by mutated procollagens and the modulation of ER stress signaling. Of interest, the anti-apoptotic component Bcl2 is down regulated during ER stress conditions attributed to *Gadd153* upregulation, leading to enhanced oxidation and apoptosis [42]. Thus, given the upregulation of *Gadd153* within *Aga2*/+ OBs, Bcl2 was likely down regulated, exacerbating and contributing to cell death. Further studies are necessary to elucidate the pro- and anti-apoptotic components that modulated the severe clinical outcome in *Aga2*/+ animals. In addition to severe long bone deformities we observed immunological, blood, heart, lung and energy metabolic defects pointing to a systemic effect of the *Col1a1* mutation. Further experiments will be necessary to elucidate the influence of the systemic defect on bone and early lethality (unpublished data).

The molecular diversity of apoptosis in OI is unknown, and it is unclear if the apoptotic program is a phenomenon of type I procollagen mutations that severely affect chain assembly and retention caused by triple helical versus C-propeptide structural defects, or by both. Recently, Forlino et al. [43] evaluated phenotype heterogeneity within the *BrtlIV* knock-in OI mouse line and identified increased *Gadd153* transcript and protein levels within calvaria of lethal animals. In this study we isolated and characterized a unique mouse model for OI, and provided evidence that the *Aga2* C-propeptide α1(I) mutation induced ER-mediated osteoblast apoptosis that affected cellular function and metabolism, which we now suggest to be a key component, among others, that influenced disease severity in Osteogenesis imperfecta.

## Materials and Methods

### Antibodies and kits.

Activated caspase-3 (R&D Systems, Germany), caspase-12 (BD Pharmingen, Germany), anti-α-tubulin (Sigma, Germany), β-actin, type I collagen, and BiP/GRP78 (all Abcam, UK), and fluorescently labeled secondary antibodies (Molecular Probes, Germany) were commercially purchased. Antisera LF-41 and -67 [44] were generous gifts provided by Dr. L. Fisher (NIH, USA). The serum biochemical analysis has been previously described [45]. The mouse TRACP 5b (IDS Ltd), osteocalcin ELISA (BTI), and mouse/rat intact PTH as well as calcitonin immunoradiometric assay (Immundiagnostik, Germany) kits were commercially purchased. All measurements were performed based on the manufacturers' recommendations.

### Animal housing and handling.

Mouse husbandry was conducted under a continuously controlled specific-pathogen-free (SPF) hygiene standard according to the Federation of European Laboratory Animal Science Associations (FELASA) protocols. Standard rodent diet and water were provided *ad libitum*. All animal experiments were conducted under the approval of the responsible animal welfare authority.

### Linkage analysis and *Aga2* genotyping.

The F_1_ dominant ENU mutagenesis screen was conducted on the inbred strain C3HeB/FeJ and has been previously described [14]. *Aga2* was backcrossed to wild-type C3H mice for five generations, and then outcrossed to C57BL/6J (F_1_) and backcrossed to the outcrossed strain (N_2_). Phenotype-positive N_2_ progeny were tail-genotyped with microsatellite markers. Recombination fractions and map distances were calculated using Mapmanager QTX. All primers for sequencing are available upon request. For genotyping, a PCR fragment containing the entire intron 50 of *Col1a1* was generated using primers for-5′-ggcaacagtcgcttcaccta-3′ and rev-5′-ggaggtcttggtggttttgt-3′. The product was then cleaved using MspA1I for gel analysis.

### Skeletal and radiological analysis.

Skeletal preparations were generated as previously described [46]. Simple and compound fractures were grossly examined. Compression fractures were evaluated using a dissecting microscope. For μCT scans (Tomoscope 10010m; VAMP, Germany), image reconstruction and visualization were performed using the ImpactCB and ImpactView software (VAMP), respectively. pQCT analysis was performed as previously described [47].

### Electron microscopy, histology, and histomorphometry.

SEM was performed as previously described [48]. For TEM, lower dorsal skin was prepared as previously described [49]*.* Sample preparation, embedding and double labeling experiments were performed as previously described [50]. For the demonstration of endogenous ALP and TRAP, the procedure of Miao and Scutt [51] was performed with modifications. All bone histomorphometric parameters were derived from the standardized nomenclature [52]. Non-sequential sections were collected every 70 - 100 μm. All images and measurements were captured and determined using an Axioplan2 workstation (AxioVision v3.1; Zeiss, Germany) and the ImageJ program (v1.36; NIH, USA). Automatic color thresholding was applied to stacked image sets. Labels were traced with minimal operator bias. 6 - 20 repeated measurements were made per section using four non-sequential sections, where *n* (4 - 6) equals the number of animals examined.

### Western analysis.

Dermal fibroblasts were cultured as previously described [53]. For caspase-12 and BiP detection, cells were lysed in RIPA buffer with protease inhibitors. Supernatants were analyzed using the NuPAGE Novex gel system (Invitrogen, UK).

### Primary OB culture and cellular assays.

OBs were prepared from 3-day-old pups with similar appearance as previously described [54]. Cells were plated at 2 × 10^4^ cells/cm^2^ for all experiments. OB differentiation was initiated via media exchange (i.e. α-MEM w/10 % FCS, 2 mM glutamine, 1 % pen-strep, 50 μg/ml ascorbic acid, 10 mM β-glycerophosphate). A minimum of four calvaria/genotype was pooled representing an independent experiment (*n*) with four repeated measurements. DNA, total protein, ALP and MTT (kit) measurements were performed in 24-well plates as previously described [55]. Caspase-3/7 activity was monitored using a fluorogenic substrate (Ac-DEVD-AMC; AXXORA, Germany) at excitation 380 nm and emission 460 nm. Mineral content was quantified by establishing an alizarin red standard curve and measuring dye release using 10 % hexadecylpyridinium in 6-well plates at 570 nm. For nodule characterization, samples were analyzed using the ImageJ software.

### Indirect immunofluorescence and TUNEL analysis.

Cells were cultured on glass coverslips coated with poly-L-lysine. For immunohistochemical analysis, sodium citrate (10 mM, pH 6.0 in microwave) and proteinase K (10 μg/ml, 37 °C) antigen retrieval methods were performed, and the Vectastain ABC kit (Vector Labs, USA) utilized. The TUNEL assay was performed with an ALP-conjugated kit (Roche, Germany). Independent preparations (*n* = 4) were analyzed with repeated measurements. For cell culture, 300 - 400 cells were counted per image field. For in vivo caspase-3 analysis, a region 0.4 mm from the growth plate was analyzed, and 400 - 480 total cells were evaluated within set regions (i.e. magnification field).

### qPCR.

RNA and cDNA were prepared as previously described [56]. qPCR was performed using the TaqMan and QuantiTect ready-to use primers (Qiagen, Germany). Duplicate crossing points per marker were averaged per independent experiment (*n* = 4). Significance was depicted between time points. Values were normalized to levels of β-actin from the same pool for fold differences.

### Statistics.

Level of significance was set at *p* < 0.5 by ANOVA test on the influence of genotype and sex, and subsequent pairwise mean comparisons performed by Student *t*- or post hoc (Bonferroni) tests. All statistics were calculated using the Prism 4 (GraphPad, USA) and SigmaStat v. 3.1 (Systat Software, Germany) software.

## Supporting Information

Figure S1 Genome-Wide Linkage and High-Resolution Haplotype Analysis of the *Aga2* Locus (A) Genome-wide linkage analysis revealed *Aga2* linkage on mouse distal Chromosome 11.
(B) Haplotype analysis identified the most probable *Aga2* candidate region between *D11Mit288* and *D11Lis2*. Log of odds ratio (LOD), centimorgan (cM), megabase (Mb), and (black) heterozygous (C3HeB/FeJ & C57BL/6 alleles), (white) homozygous (C57BL/6 allele).
(855 KB TIF)

Figure S2 In Vivo Bone Formation and Resorption in 16-Wk-Old Male Mice (A,B) Bone formation and mineral accretion were monitored by dual calcein (red arrows) and tetracycline (white arrows) in vivo labeling (dL). Tibial secondary cancellous (c, inset) and endocortical (not shown) regions were characterized. Bar, 100 μm for main in (A) and (B) combined. Bracketed bars within the corner boxes represent mean ± SEM of sample dL lengths (17.9 ± 0.6 μm/7 d, +/+; and 11.7 ± 1.0 μm/7 d, *Aga2*/+).
(C,D) Undecalcified tibial sections in control and *Aga2*/+, respectively, depict increased resorptive bays with multinucleated OCs (black arrows) at the osteogenic zone, and extended hypertrophic zone of the growth plate (Gp; horizontal arrow) in *Aga2*. Bar, 100 μm combined; stained with von Kossa and countered with Paragon. Bone marrow (Bm), trabecula (Tb).
(9.2 MB TIF)

Figure S3 Quantitative Analysis of Physiological Bone Growth and Remodeling in 16-Wk-Old Male Tibiae (A) Percentage of secondary trabecular surface encompassed with OBs.
(B) MAR calculated within both secondary cancellous (c) and endocortical (EC) bones, while BFR was calculated for mid-cancellous bones.
(C) Relative number of TRAP-positive OCs within secondary trabeculae.
(D) Cell density within femora and tibiae.
Bars represent mean ± SEM, where *n* = 4 in (A–C), and *n* = 6 experiments in (D). *p*-Values in (A–C) derived from the Student *t*-test, while in (D), *p < 0.0001 one-way ANOVA and **p < 0.001 post hoc test. Diaphysis (d), metaphysis (m), cortex (Cx), trabeculae (Tb).
(2.0 MB TIF)

Figure S4 *Aga2*/+ Osteoblastic Ex Vivo Response and Function (A) ALP activity measurement in primary OB lysates.
(B) OB total protein, reduced MTT, and secreted acid-soluble collagen levels at 21 days in culture.
(C) Time-course analysis of the density and individual area of bone-like nodules, and the amount of bounded alizarin red dye per total nodule area at 16 days. Asterisks show significant differences at *p < 0.05, **p < 0.001, and ***p < 0.0001 (Student *t*-test), while stimulated-unstimulated *p*-values were derived from ANOVA with post hoc test. Values represent mean ± SEM, where *n* = 4–5 independent experiments. 3-(4,5-dimethylthiazol-2yl)-2,5-diphenyl tetrazolium bromide (MTT), unstimulated (unstim.).
(2.4 MB TIF)

Table S1 Dysmorphology (106 KB DOC)

Table S2 In Vivo pQCT Analysis in Paternal-Derived 16-Wk-Old Male Femur (61 KB DOC)

Table S3 Clinical Chemical Analysis at 12–16 Wk of Age (54 KB DOC)

Table S4 Histomorphometry (35 KB DOC)

### Accession Numbers

*Col1a1*, MGI:88467 (murine) and HGNC:2187 (human); *Chad*, MGI:1096866; *Hspa5* (*BiP*), MGI:95835; *Serpinh1* (*Hsp47*), MGI:88283; *Ddit3* (*Gadd153*), MGI:109247. Data depicted from Mouse Genome Informatics (MGI; http://www.informatics.jax.org/) and HUGO Gene nomenclature Committee (HGNC; http://www.genenames.org).

## Abbreviations

ALP: alkaline phosphatase
BMC: bone mineral content
BMD: bone mineral density
ECM: extracellular matrix
ENU: N-ethyl-N-nitrosourea
ER: endoplasmic reticulum
OI: Osteogenesis imperfecta
UPR: unfolded protein response
