# Supplementary material for: ER Stress-Mediated Apoptosis in a New Mouse Model of Osteogenesis imperfecta
Source: PLoS Genet. 2008 Feb 1;4(2):e7. doi: 10.1371/journal.pgen.0040007 (PMC2222924; doi:10.1371/journal.pgen.0040007)
Supplement: Supplementary file 1 [file pgen.0040007.st001.doc]

**Table S1: Dysmorphology^1^**

| **Age** | **Feature** | | **+/+** | | ***Aga2*/+** | | **Pairwise** | | **ANOVA** | | |
| --- | --- | --- | --- | --- | --- | --- | --- | --- | --- | --- | --- |
|  |  |  | (A) | | (B) | | A-B  **♂** | A-B  **♀** | Geno-type | Sex | Inter-action |
|  |  |  | **♂** | **♀** | **♂** | **♀** |  |  |  |  |  |
|  |  | | *n = 15* | *n = 15* | *n = 15* | *n = 15* |  |  |  |  |  |
| 9 weeks | Abnormal gate | | 0 | 0 | 15 | 15 | *** | *** | - | - | - |
|  | Abnormal body^2^ | | 0 | 0 | 15 | 15 | *** | *** | - | - | - |
|  | Pigmented skin | | 0 | 1 | 1 | 3 | NS | NS | - | - | - |
|  | Absent/altered incisors | | 0 | 0 | 0 | 3 | NS | NS | - | - | - |
| 16 weeks | Fractured femur^3^ | | 0 | 0 | 2 | 0 | NS | NS | - | - | - |
|  | Fractured tibia | | 0 | 0 | 13 | 13 | *** | *** | - | - | - |
|  | Deformed fibula | | 0 | 0 | 7 | 6 | ** | * | - | - | - |
|  | Fractured humerus | | 0 | 0 | 6 | 9 | * | *** | - | - | - |
|  | Fractured ulna | | 0 | 0 | 10 | 9 | *** | *** | - | - | - |
|  | Fractured radius | | 0 | 0 | 5 | 7 | * | ** | - | - | - |
|  | Kyphosis/scoliosis | | 0 | 0 | 15 | 15 | *** | *** | - | - | - |
|  | Pelvis^4^ | | 0 | 0 | 15 | 15 | *** | *** | - | - | - |
|  |  | | *n = 10* | *n = 10* | *n = 10* | *n = 10* |  |  |  |  |  |
|  | Body mass [g] | | 32.14 ± 0.33 | 32.73 ± 0.96 | 21.28 ± 0.65 | 19.89 ± 0.54 | *** | *** | *** | NS | NS |
|  | Body length^5^ [cm] | | 9.95 ± 0.05 | 10.05 ± 0.08 | 8.95 ± 0.09 | 8.90 ± 0.07 | *** | *** | *** | NS | NS |
|  | Subcutaneous fat span [mm] | | 4,79 ± 0.12 | 5,41 ± 0.31 | 2.71 ± 0.12 | 3.09 ± 0.07 | *** | *** | *** | NS | NS |
|  | Vertebrae height^6^ [mg/cm^2^] | | 3.39 ± 0.05 | 3.51 ± 0.05 | 2.94 ± 0.07 | 3.09 ± 0.07 | *** | *** | *** | NS | NS |
|  | pDXA | BMD [mg/cm^2^] | 57 ± 0.1 | 60 ± 2.0 | 42 ± 1.0 | 42 ± 1.0 | *** | *** | *** | NS | NS |
|  |  | BMC [mg] | 928 ± 17 | 945 ± 46 | 307 ± 20 | 310 ± 13 | *** | *** | *** | NS | NS |
|  |  | Fat content [units x 100/g] | 30.75 ± 1.4 | 34.17 ± 2.8 | 0.63 ± 0.6 | 0.84 ± 0.8 | *** | *** | *** | NS | NS |
|  |  | Lean content [units x 100/g] | 58.19 ± 1.3 | 53.48 ± 2.9 | 93.90 ± 1.4 | 91.89 ± 1.7 | *** | *** | *** | NS | NS |
| 21 weeks | Femur length^7^ [mm] | | 15.25 ± 0.17 | 15.31 ± 0.09 | 14.61 ± 0.12 | 14.77 ± 0.15 | ** | ** | *** | NS | NS |
|  | Mediolateral shaft diameter [mm] | | 1.80 ± 0.03 | 1.75 ± 0.04 | 1.40 ± 0.03 | 1.49 ± 0.03 | *** | *** | *** | NS | ** |

^1^ Data collected from the German Mouse Clinic dysmorphology primary screen [56], direction of breeding was paternal; ^2^ slender; ^3^ healed fractures; ^4^ ischium and pubis region deteriorated; ^5^ nostril to base of tail; ^6^ third lumbar (compression); ^7^ femur length was measured as the *ex vivo* proximal (tip of greater trochanter) to distal (intercondylar fossa) distance. Measurements were made using calipers. *p* values based on Fisher’s exact test for the contingency table values, and ANOVA for the continuous variables. Data represent mean ± SEM. * *p* < 0.05, ** *p* < 0.001, *** *p* < 0.0001; NS (not significant), DXA (dual energy X-ray absorptiometry), BMD (bone mineral density, w/o head), BMC (bone mineral content).
