# Supplementary material for: ER Stress-Mediated Apoptosis in a New Mouse Model of Osteogenesis imperfecta
Source: PLoS Genet. 2008 Feb 1;4(2):e7. doi: 10.1371/journal.pgen.0040007 (PMC2222924; doi:10.1371/journal.pgen.0040007)
Supplement: Supplementary file 2 [file pgen.0040007.st002.doc]

**Table S2: *In vivo*** pQCT analysis in paternal-derived 16-week old male femur

| **Parameter & Region** | **Bone** | **+/+ (n = 6)** | **Aga2/+ (n = 6)** | **Student t test** |
| --- | --- | --- | --- | --- |
| vBMD (mg cm^-3^) |  |  |  |  |
| *Metaphysis* | Trabecular | 296.4 ± 4.46 | 200.5 ± 7.25 | *** |
|  | Cortical | 856.8 ± 6.20 | 725.5 ± 17.34 | *** |
|  | Total | 699.1 ± 8.22 | 451.1 ± 15.20 | *** |
| *Diaphysis* | Trabecular | 216.0 ± 4.62 | 172.6 ± 6.14 | *** |
|  | Cortical | 1302.0 ± 2.73 | 1198.0 ± 9.94 | *** |
|  | Total | 1034.0 ± 10.35 | 816.8 ± 16.32 | *** |
| vBMC (mg) |  |  |  |  |
| *Metaphysis* | Trabecular | 0.34 ± 0.02 | 0.30 ± 0.04 | * |
|  | Cortical | 1.95 ± 0.04 | 1.04 ± 0.06 | *** |
|  | Total | 2.28 ± 0.04 | 1.36 ± 0.06 | *** |
| *Diaphysis* | Trabecular | 0.06 ± 0.01 | 0.07 ± 0.04 | NS |
|  | Cortical | 1.79 ± 0.06 | 1.05 ± 0.06 | *** |
|  | Total | 2.02 ± 0.06 | 1.29 ± 0.07 | *** |
| Area (mm^2^) |  |  |  |  |
| *Metaphysis* | Trabecular | 1.15 ± 0.05 | 1.56 ± 0.06 | *** |
|  | Cortical | 2.27 ± 0.04 | 1.43 ± 0.07 | *** |
|  | Total | 3.41 ± 0.07 | 3.01 ± 0.08 | ** |
| *Diaphysis* | Trabecular | 0.25 ± 0.04 | 0.38 ± 0.04 | *** |
|  | Cortical | 1.38 ± 0.04 | 0.88 ± 0.05 | *** |
|  | Total | 1.96 ± 0.06 | 1.58 ± 0.07 | ** |
| Circumference (mm) |  |  |  |  |
| *Diaphysis* | Periosteum | 6.54 ± 0.07 | 6.14 ± 0.08 | ** |
|  | Endosteum | 5.04 ± 0.08 | 5.41 ± 0.10 | ** |

Values represent mean ± SEM, where n = 6 animals combining left and right femora. vBMD (volumetric bone mineral density), vBMC (volumetric bone mineral content), NS (not significant), *** *p* < 0.0001, ** *p* ≤ 0.002, * *p* = 0.116.
