# Supplementary material for: ER Stress-Mediated Apoptosis in a New Mouse Model of Osteogenesis imperfecta
Source: PLoS Genet. 2008 Feb 1;4(2):e7. doi: 10.1371/journal.pgen.0040007 (PMC2222924; doi:10.1371/journal.pgen.0040007)
Supplement: Supplementary file 3 [file pgen.0040007.st003.doc]

Table S3: Clinical chemical analysis at 12-16 weeks of age

| **Parameter** | **+/+**  (A) | | ***Aga2*/+**  (B) | | **Pairwise** | | **ANOVA** | | |
| --- | --- | --- | --- | --- | --- | --- | --- | --- | --- |
|  |  |  |  |  | A-B **♂** | A-B **♀** | Geno-type | Sex | Inter-action |
|  | **♂** | **♀** | **♂** | **♀** |  |  |  |  |  |
| *Bone Turnover* | *n = 15* | *n = 15* | *n = 15* | *n = 15* |  |  |  |  |  |
| Calcium [mmol/l] | 2.08 ± 0.03 | 2.23 ± 0.01 | 2.20 ± 0.02 | 2.21 ± 0.02 | *** | NS | ** | * | * |
| ALP [U/l) | 89.70 ± 2.73 | 137.90 ± 2.29 | 239.60 ± 8.91 | 283.39 ± 13.13 | *** | *** | *** | *** | NS |
|  | *n = 6* | *n = 6* | *n = 6* | *n = 6* |  |  |  |  |  |
| TRACP 5b[U/l] | 1.93 ± 0.04 | 2.02 ± 0.04 | 2.50 ± 0.04 | 2.40 ± 0.03 | *** | *** | *** | NS | NS |
| Osteocalcin | 227.77 ± 3.64 | 240.08 ± 3.76 | 374.30 ± 4.88 | 381.70 ± 5.15 | *** | *** | *** | NS | NS |
| *Hormones*^1^ | *n = 5* | *n = 5* | *n = 5* | *n = 5* |  |  |  |  |  |
| PTH [pg/ml] | 127.30 ± 12.69 | 107.00 ± 8.21 | 159.00 ± 8.32 | 142.40 ± 4.57 | * | * | ** | NS | NS |
| Calcitonin [pg/ml] | 31.56 ± 3.26 | 34.40 ± 2.59 | 51.26 ± 3.92 | 74.32 ± 8.12 | * | *** | *** | * | NS |

^1^ Measured at 16 weeks of age. Data presented as mean ± SEM. * *p* < 0.05, ** *p* < 0.01, *** *p* < 0.001, ALP (alkaline phosphatase), TRACP 5b (tartrate-resistent acid phosphatase 5b), PTH (parathyroid hormone), NS (not significant).
