# Supplementary material for: ER Stress-Mediated Apoptosis in a New Mouse Model of Osteogenesis imperfecta
Source: PLoS Genet. 2008 Feb 1;4(2):e7. doi: 10.1371/journal.pgen.0040007 (PMC2222924; doi:10.1371/journal.pgen.0040007)
Supplement: Supplementary file 4 [file pgen.0040007.st004.doc]

Table S4: Histomorphometry

| Indices | Structure | +/+ | *Aga2*/+ | Significance |
| --- | --- | --- | --- | --- |
|  |  | n = 6 | n = 6 |  |
|  |  |  |  |  |
| BS/TA (%) | Df (M) | 34.3 ± 4.2 | 15.9 ± 2.3 | 0.018 |
| Cx.Wi (µm) | F (Md) | 183.4 ± 5.1 | 107.0 ± 8.0 | 0.0013 |
| Tb.N (mm) | Df (M) | 4.6 ± 0.2 | 3.2 ± 0.2 | < 0.0001 |
| Tb.Th (µm) | Df (M) | 80.0 ± 7.8 | 38.5 ± 4.7 | < 0.0001 |
| Tb.Sp (µm) | Df (M) | 145.4 ± 10.0 | 281.5 ± 27.5 | 0.0001 |

Histomorphometric analysis of femora from 12-week old male mice. Values represent mean ± SEM, where averaged measurements using four nonconsecutive sections from six animals were investigated. p values were derived from the Student t test. BS/TA (bone surface area), Cx.Wi (cortical thickness), Tb.N (trabecular number), Tb.Th (trabecular thickness), Tb.Sp (trabecular separation), Df (distal femur), F (femur), M (metaphysis), Md (mid-diaphysis).
